# A Phenomenal Case of High Temperature*This article should have been published nine months ago, but it was not possible to find space for it.

**Published:** 1892-05

**Authors:** 


### A PHENOMENAL CASE OF HIGH TEMPERATURE.*

(Extract from the Minutes of the Memphis Medical Society, as reported in *Memphis Medical Monthly*, June, 1891.)

Dr. Saunders begged to hear from Dr. Jones the report of a case of wonderful temperature of which a rumor had reached him at the club.

Dr. Jones said it was his intention to report the case very fully and minutely later on in connection with Dr. Sale, who was treating it with him, as he thought it fully justified such a report. At present he would present the following brief statement of it :

The patient was a bright girl, fourteen years old. Three

---

* This article should have been published nine months ago, but it was not possible to find space for it.

weeks ago she had an attack of tonsillitis, from which she had suffered frequently before. The relative with whom she was staying was a lady of intelligence, and an experienced nurse. She reported to Dr. Jones temperature of 103° or 104° or 105°. As he was never at his visits able to find any fever, he thought there must be some error. One day she telephoned that the temperature was 108°. He hurried down and found it 109°. The tonsillitis was about well. He was alarmed, and sent for Dr. Sale. Directly it declined. The next day the family reported that the index had gone to the top of the thermometer. The thermometer was graduated to 112°, with room above for perhaps 2° more. He went at once and found temperature 97°. The same day it again went to the top in the space of two or three minutes. For two or three days it reached this height several times daily. Then, two weeks ago, the heat began breaking the thermometers, the mercury being expanded beyond the capacity of the bulb and tube. This occurred in the axilla and in the mouth, the break occurring sometimes at the bulb, sometimes along the tube and in plain view, so not from muscular action or contact with teeth. Eight thermometers have been thus broken. [The doctor exhibited two, the index in both cases standing at the top of the tube, 114° in one and 115° in the other.] The people are intelligent and honest, and there is no ground of suspicion or deception.

Dr. Sale, even by all this, had not been convinced, and ordered a thermometer to register 150°. While this thermometer is graduated to 150°, the index may register 9°, at least 6° higher. The first observation with this showed a normal temperature; on the second observation it registered 115°, at the third 135°. During the same night it reached 150°. Last night the mercury reached the top of the tube, but reaching there directly after, he found it 99½°. Dr. Neely saw the patient during the day, but his visit was during a period of defervescence.

Last night in the axilla the index reached the top, and being again placed in the axilla the expanding mercury burst the thermometer.

The rises of temperature occur several times a day, and are very rapid.

A peculiar feature was that the patient recognized with great certainty the rise and fall of the temperature. The rise was accompanied by a peculiar sensation of the face, a benumbing, and the patient would indicate the grade of the fever by the terms, numb, number, numbest. Usually, when the temperature was observed rising, the hands and feet would be found cold.

There is no possibility of deception; the people are thoroughly trustworthy, and are horrified at the high temperature, and the girl herself is greatly alarmed.

The circulation is never accelerated beyond 10 to 15 beats to the minute, being usually 80. It has not recently been above 100, nor at any time beyond 120. When the pyrexia is greatest the condition becomes alarming; hands and feet are cold, the surface covered wih clammy sweat; there is severe nausea, and the patient expresses herself as feeling bad. These symptoms go with the fever, and cheerfulness and well-being return, and where there is freedom from high temperature the girl has no appearance of being very sick. She is pallid, but her strength is good.

The pupils of the girl's eyes are generally normal, but at times she can dilate or contract them at pleasure, as an owl or parrot. With the hyperpyrexia there is always nausea and a sense of oppression in the chest.

Incidentally it may be stated that the girl is an athlete, or contortionist, excelling in running, jumping, etc., can place her feet behind her head, and get into other incongruous shapes.

Dr. Jones attempts no explanation of the condition. When the temperature began, he supposed it due to malaria, and gave large doses of Quinine for three days.

The paroxysms were not regular. In the last twenty-four hours there were four rises of temperature, lasting never beyond three or four hours, usually much less time. The family keep an accurate record of temperature.

